# Isolation, Identification, and Genomic Characterization of Chicken Astrovirus Isolates From China

**DOI:** 10.3389/fvets.2021.800649

**Published:** 2022-02-22

**Authors:** Wei Zhao, Jialei Shi, Yongxiu Yao, Hongxia Shao, Aijian Qin, Kun Qian

**Affiliations:** ^1^Ministry of Education Key Lab for Avian Preventive Medicine, Yangzhou University, Yangzhou, China; ^2^Jiangsu Key Lab of Preventive Veterinary Medicine, Yangzhou University, Yangzhou, China; ^3^The Pirbright Institute & UK-China Centre of Excellence for Research on Avian Diseases, Surrey, United Kingdom; ^4^Jiangsu Co-innovation Centre for the Prevention and Control of Important Animal Infectious Diseases and Zoonoses, Yangzhou University, Yangzhou, China

**Keywords:** astrovirus, chicken, isolation, full-length genome sequence, molecular characterization

## Abstract

Chicken astrovirus (CAstV) infection can cause diarrhea, nephritis, stunted growth, and “white chickens” condition, resulting in economic losses to the poultry industry. Currently, a few CAstVs were isolated and a few full-length genome sequences of CAstV have been deposited in the GenBank. In the present study, two CAstV isolates (AAstV/Chicken/CHN/2017/NJ01 and AAstV/Chicken/CHN/2018/CZ01) were successfully isolated by using LMH cells, and we molecularly characterized these two CAstV isolates and observed the effect of these two isolates on hatchability using chicken embryo infection experiment. The genetic analysis demonstrated that these two strains had the typical characteristics of avian astroviruses, which were composed of three open reading frames, 5′UTR, and 3′UTR. The full-length genome sequence showed a high-degree identity at nucleotide level of 97.5–98.7% among Chinese isolates suggesting their common ancestors and limited sequence divergence. Sequence analysis of ORF2, which encodes the capsid protein associated with classification of avian astrovirus, revealed our two isolates belonging to CAstV Bi subtype. At the amino acid level, the complete capsid region of Chinese strains shared genetic distances of 0.03–0.04 with FP3 strains isolated from the UK, suggesting their common origin. Meanwhile, hatchability reduction was observed. These results provided novel insights into the molecular epidemiology and hatchability effect of CAstV.

Chicken astroviruses (CAstV) are non-enveloped, single-stranded RNA viruses belonging to the family *Astroviridae* (1, 2). The genome of CAstV is composed of 5′UTR, three open reading frames (ORF1a, ORF1b, and ORF2), 3′UTR, and Poly (A) tail. Among them, ORF1a and ORF1b encode nonstructural proteases, which are related to viral transcription and replication. ORF2 is a highly variable region of the genome, encoding capsid protein, which is the main protein inducing host immune response in the immune response process (3, 4). As reported previously, there are two classification methods for CAstV. One is based on capsid protein of CAstV, which can be divided into type A and type B (5). Another conservative method is to classify CAstV into type I and type II CAstV according to the RNA-dependent RNA polymerase (RdRp) gene encoded by ORF1b (6).

CAstV was first isolated and genomic characterized in 2004 from two flocks of broilers in the UK (5). According to previous reports, the diversity of CAstV and cross-species transmission between turkey and chicken deserve our attention (2, 7). In recent years, the presence of CAstV has been detected in chicken flocks from different provinces of China, and both type I and type II CAstV can exist in the same flock (8–10). In order to further understand the genetic characteristics of CAstVs circulating in China, we sequenced and compared the whole genome of two CAstV strains deposited in GenBank, CAstV-NJ1701 (accession no. MK746105.2) and CAstV-CZ1801 (accession no. MN807051.1), isolated from Nanjing and Changzhou of Jiangsu province in 2017 and 2018. These chickens have the clinical symptoms of growth and development disorder syndrome, and diarrhea. As described in our previous report (10), the homogenate of ceca samples of 1-day-old broilers were filtered through a 0.22-μm filter, and the 0.5-ml filtrate was inoculated onto LMH (chicken hepatocellular carcinoma epithelial cell line, CRL-2117, ATCC) cultures in six-well plates. After incubation for 3 h, the supernatant was replaced with normal LMH cell culture medium. The cells underwent three freeze–thaw cycles after incubation for 3 days, and the supernatant was directly inoculated onto new LMH cultures. The AAstV/Chicken/CHN/2017/NJ01 (CAstV NJ1701) and AAstV/Chicken/CHN/2018/CZ01 (CAstV CZ1801) isolates were obtained by the fifth passage in LMH cells with obvious cytopathic effect (data not shown).

The viral genes were detected by quantitative reverse transcriptase polymerase chain reaction (qRT-PCR). Then the two isolates were purified further using plaque purification assay (data not shown). After confirmation of the purified CAstV and ruling out the presence of other common enteric viruses by RT-PCR as described previously (10), the viral RNAs were extracted for next-generation sequencing using an Illumina MiSeq System (Illumina Inc., San Diego, CA, USA) by Tanpu Biotechnology Company, Shanghai. The resulting sequence was BLAST searched in GenBank for similar sequences. Based on the Jones–Taylor–Thornton model and 1,000 bootstrap repeats, the neighbor-joining method of MEGA X was used to perform phylogenetic tree analysis. The inheritance of amino acid distance was calculated by *p*-distance method of MEGA X.

The whole-genome sequences of two isolates, CAstV NJ1701 and CAstV CZ1801, were successful1y obtained. According to the sequence analysis and alignment, these two isolates had the same length of genome, three open reading frames (ORFs), but different length in two untranslated regions (UTR) (Figure 1A). The sequences of the nearly full-length genome of these two CAstV strains consisted of 7,603 nt, including ORF1a, ORF1b, ORF2, 5′UTR, and 3′UTR sequences, excluding the poly(A) tail. The genome was 15 nt longer than the genome sequence of the deposited Chinese strain CAstV/CHN/HBLP717/2018 (accession no. MN725025), and 13 nt longer than the other Chinese strain CAstV/CHN/GDYHTJ718-6/2018 (accession no. MN725026). The 5′UTR and 3′UTR sequences were 69 and 335 nt in CAstV NJ1701, and 76 and 328 nt in CAstV CZ1801, respectively. These two CAstV strains had the typical structure of Astrovirus with three overlapping ORFs (Figure 1A). As shown in Figure 1B, ORF1a contained 3,420 nucleotides and encoded 1,139 amino acids (aa). In the overlapping region of ORF1a and ORF1b (nt 3,471 to 3,489 in CAstV NJ1701 and nt 3,478 to 3,496 in CAstV CZ1801), the seven-base heptameric frameshift sequence (AAAAAAC) containing the ribosome frameshift signal (RFS) was present. ORF1b contained 1,560 nucleotides, encoding RNA-dependent RNA polymerase with 519 aa. A typical 24-nt spacer between ORF1b and ORF2 at positions 5,030 to 5,055 in CAstV NJ1701 and 5,037 to 5,062 in CAstV CZ1801 were also observed (Figure 1B). ORF2 contained 2,214 nucleotides, encoding the capsid protein of 737 aa. The 3′UTR region contained two stem-loop structures predicted by the s2m motif, which were located at positions 7,245–7,287 and 7,340–7382 in CAstV NJ1701, and 7,252–7,294 and 7,347–7,389 in CAstV CZ1801, respectively. The exact role of the stem-loop structure is not clear, but some studies described it as a genetic element, which could affect gene expression in the infected organisms by RNA interference (11, 12).

**Figure 1:**
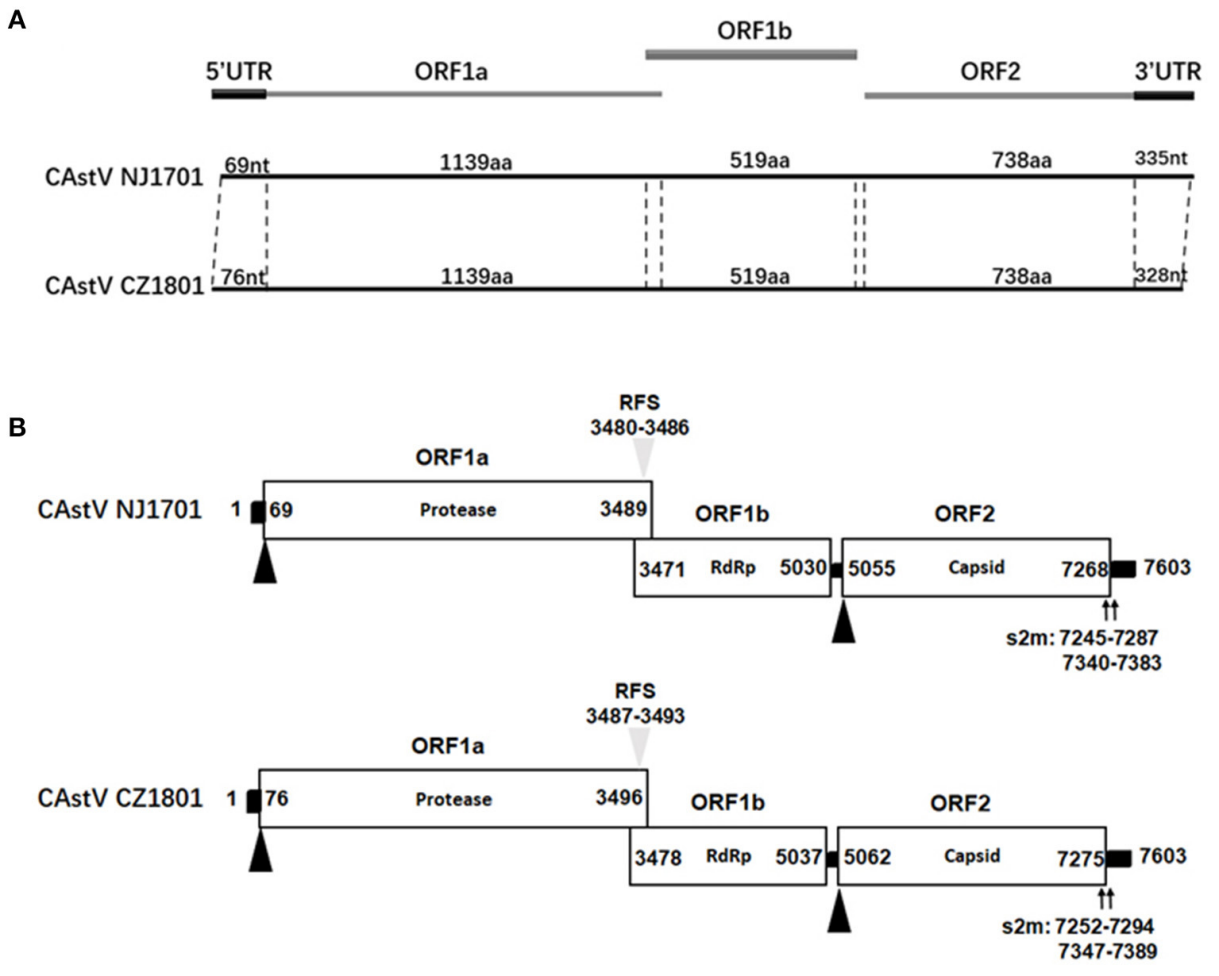
**(A)** Genome organization of chicken astrovirus isolates chicken astroviruses (CAstV)/NJ1701 and CAstV/CZ1801. **(B)** Three open reading frames (ORFs) with their locations and the motifs typical for astroviruses are shown. The translation start sites of ORF1a and ORF2 are indicated by black triangles. The nucleotide position of the start site of the heptameric AAAAAAC (RFS) sequence is shown by gray triangles. Black bars are the untranslated regions and the 24-nt spacer between the stop and start codons of ORF1b and ORF2, respectively. ORF, open reading frame; RdRp, RNA-dependent RNA polymerase; RFS, ribosomal frameshift signal; s2m, stem-loop-II-motif.

A comparison with the genomic characteristics of the two isolates with other AAstVs were then conducted (Table 1). The nearly full-length genome sequence of CAstV/CZ1801 had the closest similarity to those of Chinese CAstV/NJ1701, CAstV/HBLP, and CAstV/GDYHTJ, at the level of 97.5–98.7%. Except for Chinese CAstVs, the homology of CAstV/CZ1801 to the current published CAstV full-length genome sequences in GenBank was 69.0–80.4% (Table 1). The amino acid sequences of ORF1a and ORF1b of CAstV/CZ1801 shared the highest identities of 98.4 to 99.6% with the published sequences of Chinese CAstVs as well (Table 1). The amino acid sequence similarity of ORF2 to these avian astrovirus strains published in GenBank ranged between 26.6 and 99.9%. Interestingly, the ORF2 sequence of the UK isolate CAstV/FP3 had a high similarity with CAstV/CZ1801 at the amino acid level, reaching 95.9%, and the ORF2 of the other three Chinese strains had very high homology with FP3 also. The genetic distances of the amino acid sequence of ORF2 between CAstV/CZ1801, CAstV/NJ1701, CAstV/HBLP717, CAstV/GDYHTJ718, and the UK strain CAstV/FP3 were from 0.03 to 0.04, respectively (Table 1), indicating that they might have a common origin. However, we cannot further analyze the relationship between the Chinese strains and the FP3 strain as the full-length genome sequence of CAstV/FP3 is lacking.

**Table 1:** Comparisons of nucleotide and amino acid sequences of CAstV/CZ1801 with selected representative astroviruses.

**Species**	**Virus**	**GenBank** **accession no**.	**Sequence identity (%)**			**Genetic** **distance**	
			**Genome** **(nt)**	**ORF1a** **(aa)**	**ORF1b** **(aa)**	**ORF2** **(aa)**	**ORF2** **(aa)**
	CAstV/NJ1701	MK746105.2	97.7	99.5	99.6	99.9	0.03
	CAstV /HBLP	MN725025.1	98.7	99.3	99.2	99.9	0.03
	CAstV/GDYHTJ	MN725026.1	97.5	98.4	99.4	99.7	0.04
CAstV	CAstV/INDIA	KY038163	80.4	87.0	86.1	90.6	0.09
	CAstV CA-AB	MT789775	78.3	86.7	86.3	88.5	0.09
	CAstV CA-SK	MT789787	78.2	86.9	87.3	87.8	0.09
	CAstV/GA2011	JF414802	79.7	86.8	86.9	84.2	0.16
	CAstV/CC	ARI45593	78.1	86.9	86.9	88.3	0.12
	CAstV/CkP5	ARI45596	78.1	86.9	86.9	88.5	0.12
	CAstV/Poland/G059	KT886453	69.0	86.4	86.1	39.5	0.62
	CAstV/4175	JF832365	75.6	86.4	77.9	80.3	0.20
	CAstV/FP3	JN582328	–	–	–	95.9	0.04
	CAstV/P22-18.800	AFK92941	–	–	–	38.9	0.6
	CAstV/VF08-29	AFK92938	–	–	–	84.1	0.16
	CAstV/612	AFK92940	–	–	–	38.8	0.61
	CAstV/VF08-48	AFK92949	–	–	–	39.8	0.60
	CAstV/VF08-56	AFK92942	–	–	–	38.9	0.60
TAstV	TAstV-1	Y15936	44.3	40.7	57.3	37.2	0.64
	TAstV-2	AF206663	51.0	46.3	69.5	39.2	0.63
DAstV	DAstV/C-NGB	FJ434664	57.4	57.3	71.5	38.4	0.63
	DAstV/SL1	KF753804	55.0	47.4	72.2	37.3	0.64
GAstV	GAstV/GD	MG934571	54.6	47.9	63.9	37.6	0.64
ANV	ANV-1/China	HM029238	47.2	26.7	53.2	26.6	0.75

*ORF, open reading frame; aa, amino acids; CAstV, chicken astroviruses*.

Phylogenetic analysis of the complete aa sequence revealed that CAstV/CZ1801 and CAstV/NJ1701 were in the same branch as the other two Chinese strains, CAstV/HBLP717 and CAstV/GDYHTJ718 (Figure 2A). The similar phylogeny based on amino acid sequence were obtained for ORF1a (Figure 2B) and ORF1b (Figure 2C). In order to get further insight into the evolutionary relationship of the two isolates in this study with other Avastroviruses, the phylogenetic analysis based on the amino acid sequence of full-length ORF2 was performed. According to a previous report, phylogenetic analysis based on the complete capsid gene sequences of chicken astroviruses demonstrated the existence of two major capsid groups, designated as A and B (5, 6). The two isolates CAstV/CZ1801 and CAstV/NJ1701, along with other two strains CAstV /HBLP717 and CAstV/GDYHTJ718 from China were found to be in group Bi with CAstV/FP3 (Figure 2D).

**Figure 2:**
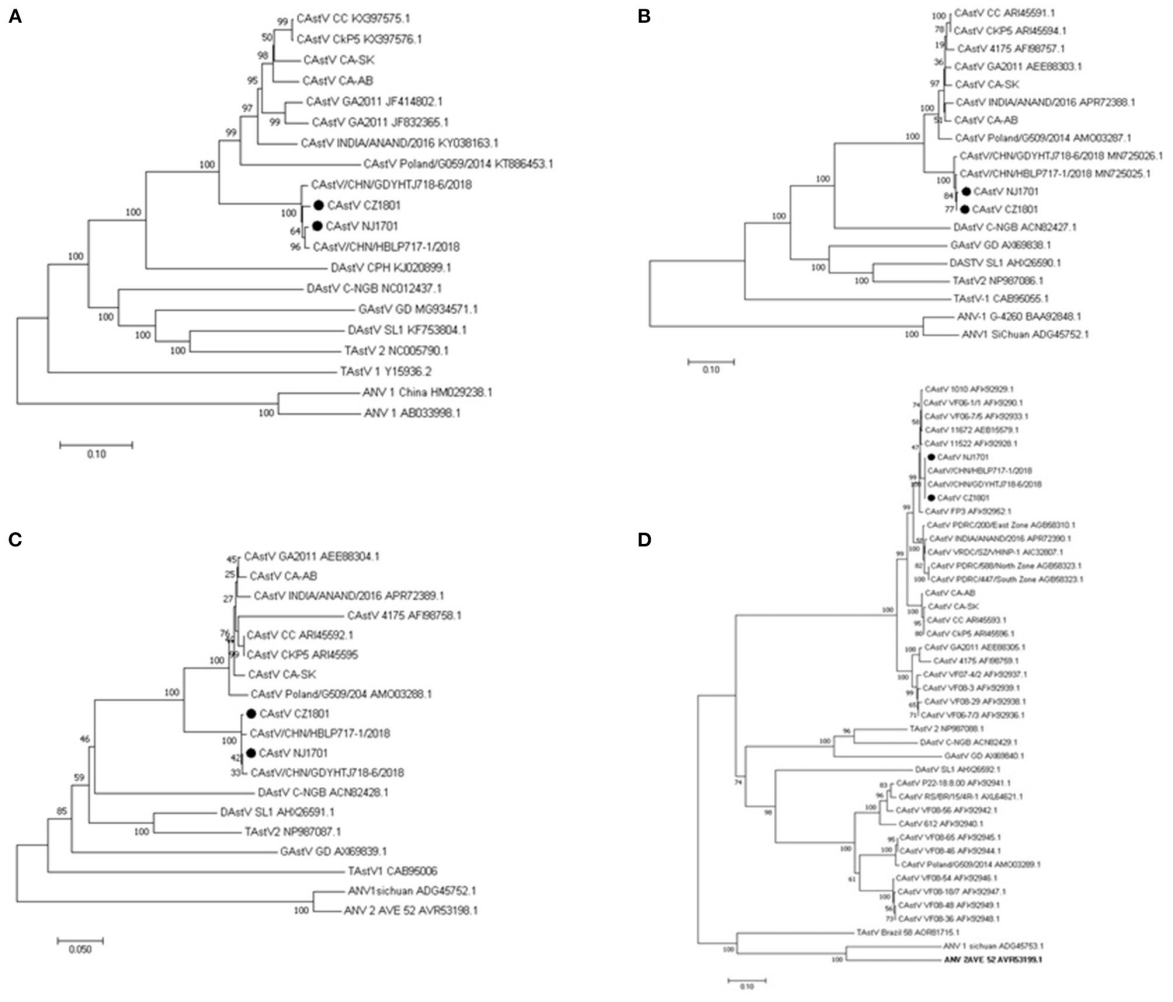
Phylogenetic relationships between the two CAstV isolates from this study and other astroviruses. The analysis was based on the nucleotide sequences of the complete genome **(A)** and on amino acid sequences of the complete ORF1a **(B)**, ORF1b **(C)**, and ORF2 **(D)** regions. The phylogenetic trees were constructed using MEGA X, the neighbor-joining method and 1,000 bootstrap replicates. GenBank accession number of the sequences are indicated in parentheses. The scale bar corresponds to genetic distance. “•” indicates the isolates analyzed in this study.

According to our previous published study (10), SPF chicken embryos were used for investigating the effect of two CAstV isolates on chick hatching rate. The hatching rate of CAstV/NJ1701 infection was 10/30 (33.33%), which significantly decreased when compared with normal control group. However, the hatching rate 26/30 (86.67%) of CAstV/CZ1801 infection only slightly decreased. These results suggested that these two strains might have different pathogenesis.

Taken together, two CAstV strains, CAstV/CZ1801 and CAstV/NJ1701, were isolated by using LMH cells. Both of the isolates reduced the hatchability of the chicken embryo. The results of full-length genome analysis showed that the two isolates had little variation compared with other Chinese isolates, but were significantly different from those isolated from other countries. The present study contributes to the understanding of the epidemiology and diversity of chicken astrovirus infection in China. Although the hatchability reduction was observed in this study, the pathogenicity of the viruses in chicken needs further investigation to elucidate.

## Data Availability Statement

The datasets presented in this study can be found in online repositories. The names of the repository/repositories and accession number(s) can be found in the article.

## Ethics Statement

The animal study was reviewed and approved by the Animal Care Committee of Yangzhou University.

## Author Contributions

KQ and AQ designed the study. WZ and JS carried out the experiments, analyzed the data, and drafted the manuscript. KQ supervised all the experiments and participated in the data analysis. YY, HS, and AQ discussed and revised the final manuscript. All authors contributed to the article and approved the submitted version.

## Funding

The research was supported by the NCFC-RCUK-BBSRC (Grant No. 31761133002), BBSRC Newton Fund (BB/R012865/1), Foundation of Cultivate Middle-aged and Young Science Leaders of Colleges and Universities of Jiangsu Province, the Priority Academic Program Development of Jiangsu Higher Education Institutions.

## Conflict of Interest

The authors declare that the research was conducted in the absence of any commercial or financial relationships that could be construed as a potential conflict of interest.

## Publisher's Note

All claims expressed in this article are solely those of the authors and do not necessarily represent those of their affiliated organizations, or those of the publisher, the editors and the reviewers. Any product that may be evaluated in this article, or claim that may be made by its manufacturer, is not guaranteed or endorsed by the publisher.
